# Dysgonomonas mossii Strain Shenzhen WH 0221, a New Member of the Genus *Dysgonomonas* Isolated from the Blood of a Patient with Diabetic Nephropathy, Exhibits Multiple Antibiotic Resistance

**DOI:** 10.1128/spectrum.02381-21

**Published:** 2022-08-01

**Authors:** Xiaojuan Gao, Jiehong Wei, Tongyu Hao, Ting Yang, Xinyuan Han, Mengmeng Li, Xinying Li, Dan Xiong, Xiuming Zhang

**Affiliations:** a Medical Laboratory of Shenzhen Luohu People's Hospital, Shenzhen, Guangdong, China; Johns Hopkins Hospital

**Keywords:** *Dysgonomonas mossii*, species identification, multiple antibiotic resistance, whole genome sequencing

## Abstract

Herein, we present a new bacterial strain isolated from infected blood of a patient with diabetic nephropathy. Matrix-assisted laser desorption/ionization time-of-flight mass spectrometry failed to identify the strain. 16S rRNA gene sequencing showed the highest similarity (>99.5%) with genus *Dysgonomonas*, but the strain could not be distinguished from Dysgonomonas oryzarvi and Dysgonomonas mossii. Whole genome sequencing, followed by phylogenetic analysis and average nucleotide identity (>95%) analysis, confirmed that the new strain represented Dysgonomonas mossii, leading it to be named Dysgonomonas mossii strain Shenzhen WH 0221. Shenzhen WH 0221 was 3.60 Mb with 37.4% GC content. It was Gram-stain negative, facultatively anaerobic, and grown on Columbia agar supplemented with 5% of sheep blood, exhibiting a smooth surface and pinpoint morphology. The morphological characteristics of this strain include a short rod shape without flagella and a size of 0.45–0.55 × 0.95–1.52 μm observed under transmission electron microscopy. The physiological and biochemical features and major cellular fatty acids (characterized by C_14:0_ 3-OH, C_14:0_ 9-CH_3_, and C_16:0_) differed from *D. mossii* CCUG 43457^T^ and other members of the genus *Dysgonomonas*. The isolate was found resistant to most cephalosporins, penicillin, norfloxacin, vancomycin, and chloramphenicol, but was susceptible to meropenem, imipenem, tetracycline, clindamycin, and amoxicillin-clavulanic acid. Genes *kdpE*, *ykkD*, *cmeB*, *TLA-3*, and *vanRM* found in its genome are probably associated with multiple antibiotic resistance. Lipopolysaccharides, capsules, and cytolysin may also help to illuminate its potential pathogenicity. This is the first report of a case of sepsis caused by Dysgonomonas mossii, and its pathogenic system was analyzed by whole genome sequencing.

**IMPORTANCE** This study identified a new strain, Dysgonomonas mossii strain Shenzhen WH 0221, which has been first reported to cause sepsis isolated from infected blood of a patient with diabetic nephropathy. Physiological and biochemical characterizations, as well as overall fatty acid profile, distinguish Shenzhen WH 0221 from other species of the same genus. However, limited antibiotics were researched for Dysgonomonas mossii. Seventeen antibiotics spanning at least 6 classes were studied, providing a valuable guide to the clinical usage of drugs to treat Dysgonomonas mossii infection. For the first time, we report genome-based functional predictions for Dysgonomonas mossii. Five antibiotic resistance ontologies and more than 200 virulence factors likely underlie the multidrug resistance of Shenzhen WH 0221 and its potential pathogenicity.

## INTRODUCTION

The members of genus *Dysgonomonas* are Gram-stain negative, facultatively anaerobic, coccobacillus-shaped bacteria within the phylum Bacteroidetes, class Bacteroidia, and order Bacteroidales (1). Genus *Dysgonomonas* was established by Hofstad (2) from what was formerly designated as CDC (Centers for Disease Control) group DF (Dysgonic fermenter)-3 organisms (3–5). Several species within the genus *Dysgonomonas* have been recognized as polyphyletic based on 16S rRNA sequence. Nine species of the genus *Dysgonomonas* have been identified so far: Dysgonomonas capnocytophagoides (2), Dysgonomonas gadei (2), *Dysgonomonas mossi* (6), Dysgonomonas hofstadii (1), Dysgonomonas oryzarvi (7), Dysgonomonas macrotermitis (8), Dysgonomonas alginatilytica (9), Dysgonomonas termitidis (10), and *Dysgonomonas massiliensis* (11). The first four species have been isolated from human sources, including urine (3), intestine (12), blood (13), gallbladder (2), abdominal wounds (1), and abscesses (14). Dysgonomonas oryzarvi was detected in field soil relevant to the development of microbial fuel cells. Dysgonomonas macrotermitis and Dysgonomonas massiliensis are isolated from the guts of different animals, including termites (10), fruit flies (15, 16), red palm weevils (17), and European seabass (18). According to the available reports of *Dysgonomonas* isolated from human sources, organisms of genus *Dysgonomonas* are likely to infect immunocompromised patients with severe underlying diseases (12, 14).

In this article, we report the first isolation in China of Dysgonomonas mossii from the blood of a patient diagnosed with pulmonary infection, type 2 diabetes, kidney disease, and essential hypertension while in our hospital. To date, sepsis cause by isolated strains from the genus *Dysgonomonas* has not, to our knowledge, been reported. The advent of next-generation sequencing and comparative genomics analysis has greatly facilitated the study of pathogenic mechanisms. In addition to morphological characteristics, we systematically assessed the resistance phenotypes, whole genome-based phylogeny and taxonomy, resistant mechanisms, and virulence genes of the new isolate.

## RESULTS

### Strain isolation and characterization.

The strain Shenzhen WH 0221 was originally isolated from a human clinical source, two sets of blood specimens cultured anaerobically at Luohu People's Hospital, Shenzhen, China. The patient was a 78-year-old female with severe underlying diseases, including Stage 3 diabetic nephropathy, primary hypertension, and intramuscular vein thrombosis of the right lower extremity. She was admitted to the ICU due to cough and pulmonary infection. Shenzhen WH 0221 was a facultative anaerobic organism isolated from blood cultures. After 24 h of incubation at 37°C, colonies on Columbia Blood Agar were semitransparent, less than 1 mm in diameter and had a smooth surface.

**FIG 1:**
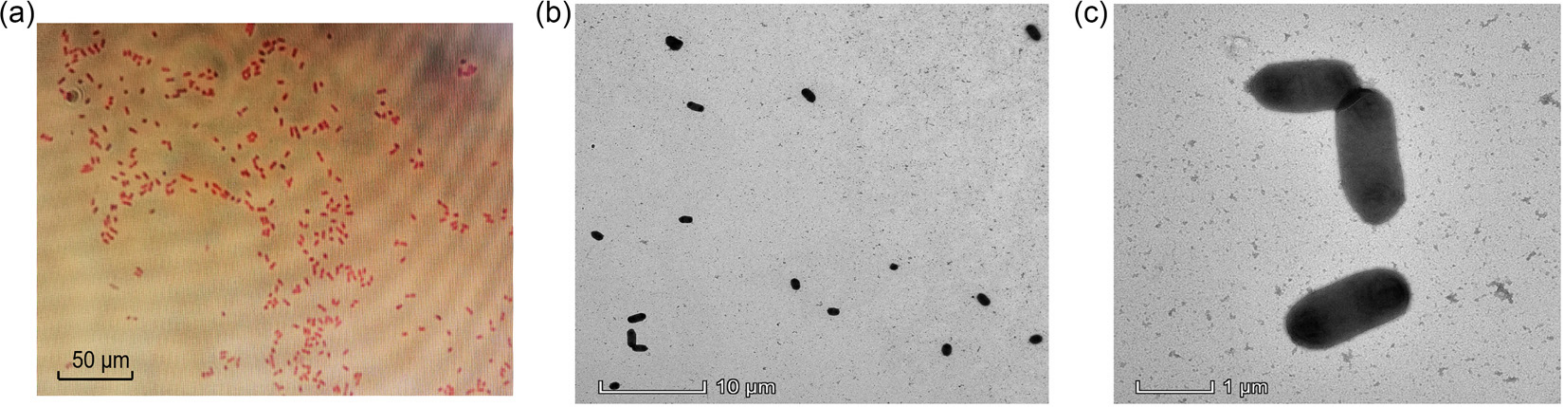
Morphology of Dysgonomonas mossii strain Shenzhen WH 0221. (a) Gram-staining was performed on the strain Shenzhen WH 0221 and then observed under the inverted microscope (magnification, ×1000). (b, c) The ultrastructure of cells of strain Dysgonomonas mossii strain Shenzhen WH 0221 was scanned using TEM. Bars, 50 μm (a), 10 μm (b), 1 μm (c).

The morphology of the new isolate was analyzed by Light Microscopy and transmission electron microscopy (TEM). Cells were Gram-negative and rod-shaped (Fig. 1a). TEM showed that Shenzhen WH 0221 cells were 0.45–0.55 × 1.20–1.52 μm in size and coccobacillus-shaped (Fig. 1b and c). Its shape was consistent with previously described species in the genus *Dysgonomonas* (1). Also, no flagellum or sporulating was observed under TEM (Fig. 1b and c). Shenzhen WH 0221 was initially subjected to MALDI-TOF MS, but the identification failed as the confidence value was too low. Then, the Vitek 2 anaerobic and corynebacteria (ANC) identification card and Vitek 2 Gram-Negative Identification (GN) card were used to identify the organism. The former showed Unidentified Organisms, and the latter recognized the organism as *Sphingonas paucimobilis*. To conform this result, 16S rRNA gene sequence determination was performed.

### 16S rRNA gene sequencing and phylogenetic analysis.

**FIG 2:**
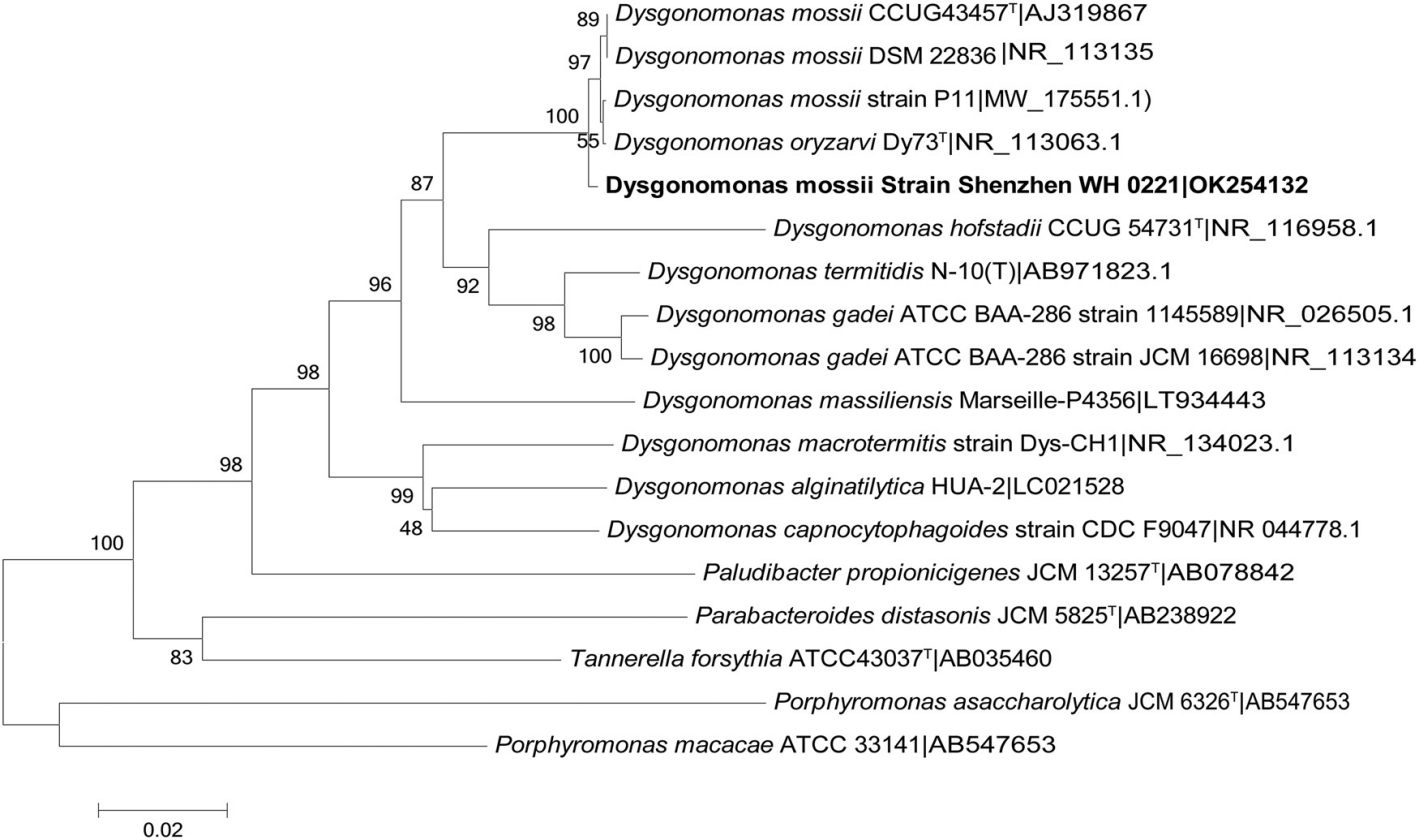
The phylogenetic tree based on the 16S rRNA gene sequence representing the position of the Dysgonomonas mossii strain Shenzhen WH 0221 (GenBank number: OK254132) and other closely related species. Bootstrap values were shown at the branch points based on 1,000 resamplings. Bar, 0.02 substitutions per nucleotide position.

A partial 16S rRNA gene sequence (1,363 bp) was acquired by Sanger Sequencing. Sequence analysis revealed >99.5% similarity to five strains of the genus *Dysgonomonas*: Dysgonomonas oryzarvi strain CBA7536, *Dysgonomonas* sp. AM15, *Dysgonomonas* sp. WJDL-Y1, Dysgonomonas mossii strain P11-biofilm, and *Dysgonomonas* sp. A1 (Table S1 in the supplemental material). The sequences of nine species from genus *Dysgonomonas* were downloaded from NCBI, and a phylogenetic tree was constructed based on 16S rDNA (Fig. 2). Shenzhen WH 0221 was clustered in the genus *Dysgonomonas.* Therefore, the newly isolate was identified as a member of genus *Dysgonomonas*. However, based on 16S rDNA alone, we could not confirm whether it belonged to Dysgonomonas mossii or Dysgonomonas oryzarvi.

### Whole genome sequencing and genome related indices.

**FIG 3:**
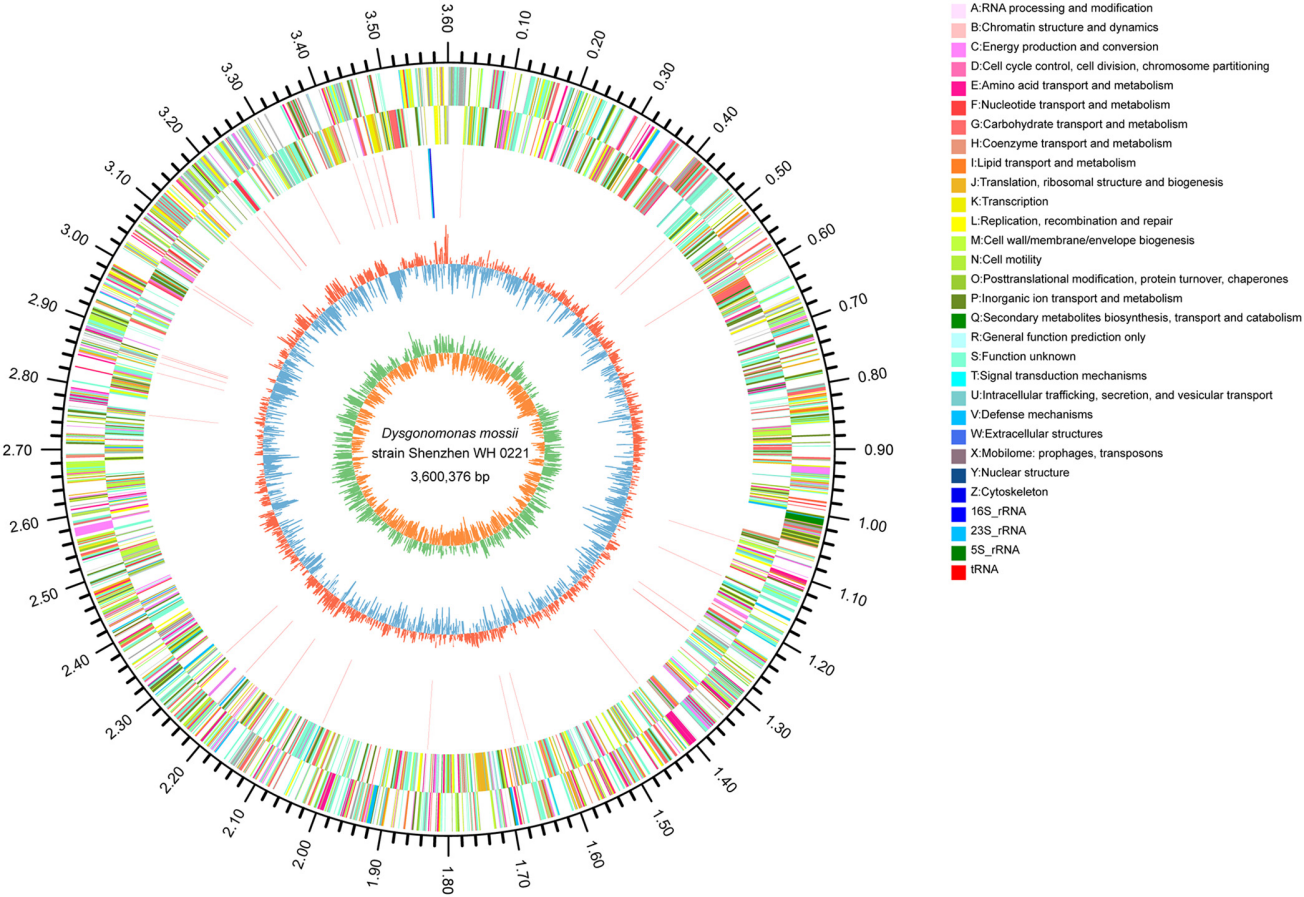
Circular maps of Dysgonomonas mossii strain Shenzhen WH 0221. Circles are numbered from the outermost (first) to the innermost (sixth) circle and include the following features: scale in Mb (first circle); coding DNA sequences on forward chain with different colors based on COGs categories (second circle); coding DNA sequences on reverse chain with different colors based on COGs categories (third circle); rRNA and tRNA (fourth circle); GC content (fifth circle); GC skew (sixth circle).

The genome of strain Shenzhen WH 0221 was sequenced in this study. Quality control of whole-genome sequencing data is shown in Table S2. The genome of strain Shenzhen WH 0221 comprised a total length of 3,600,376 bp with a G+C content of 37.41% and 3,124 coding sequences. It contained 3 rRNA genes and 44 tRNA genes (Table 1). A circular map of Dysgonomonas mossii strain Shenzhen WH 0221 is shown in Figure 3.

**TABLE 1:** General features of the genome sequences of genus *Dysgonomonas*

Organism	Strain	Size（Mb）	G+C (%）	CDS^*a*^	rRNA	tRNA
Dysgonomonas mossii	Shenzhen WH 0221	3.60	37.4	3124	3	44
Dysgonomonas mossii	P11	4.16	37.6	3538	11	41
Dysgonomonas mossii	DSM 22836	3.95	37.4	3359	15	46
Dysgonomonas mossii	MGYG-HGUT-01377	3.95	37.4	3359	15	46
Dysgonomonas alginatilytica	DSM 100214	5.12	36.7	4235	9	43
Dysgonomonas capnocytophagoides	DSM 22835	4.38	37.7	3496	13	45
Dysgonomonas gadei	ATCC BAA-286	5.18	39.5	4080	13	38
Dysgonomonas massiliensis	Marseille-P4356	3.47	37.3	2951	7	46
Dysgonomonas sp.	BGC7	4.32	37.1	3511	3	39

a CDS, coding sequence.

**FIG 4:**
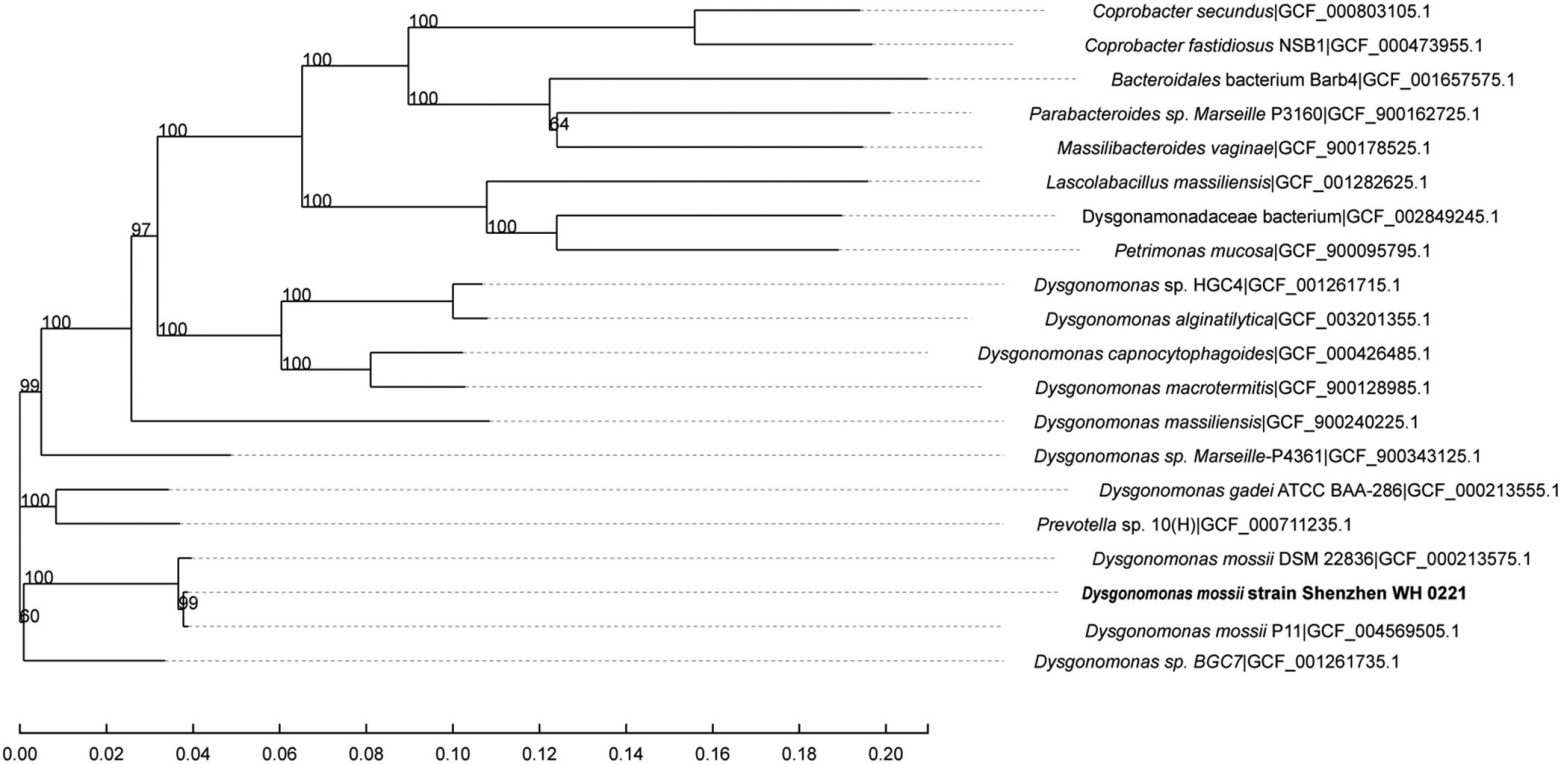
The phylogenetic tree based on 31 housekeeping genes of the type strains of the type species closest to strain Shenzhen WH 0221. The tree was constructed using the neighbor-joining method. The numbers at the nodes indicate the bootstrap values (in percentage) from 1,000 replicates.

The complete sequences of 31 housekeeping genes were extracted from genomic sequences of the strain Shenzhen WH 0221 and from 19 closely related strains. These housekeeping genes showed sequence similarity values ranging from 77.1 to 99.8% (Table S3). A multilocus sequence analysis (MLSA)-based phylogenetic tree showed that strain Shenzhen WH 0221 is in the same clade as Dysgonomonas mossii P11(similarity value: 99.8%) and Dysgonomonas mossii DSM22836 (similarity value: 99.4%) (Fig. 4). These results suggest that strain Shenzhen WH 0221 belongs to the species Dysgonomonas mossii.

**FIG 5:**
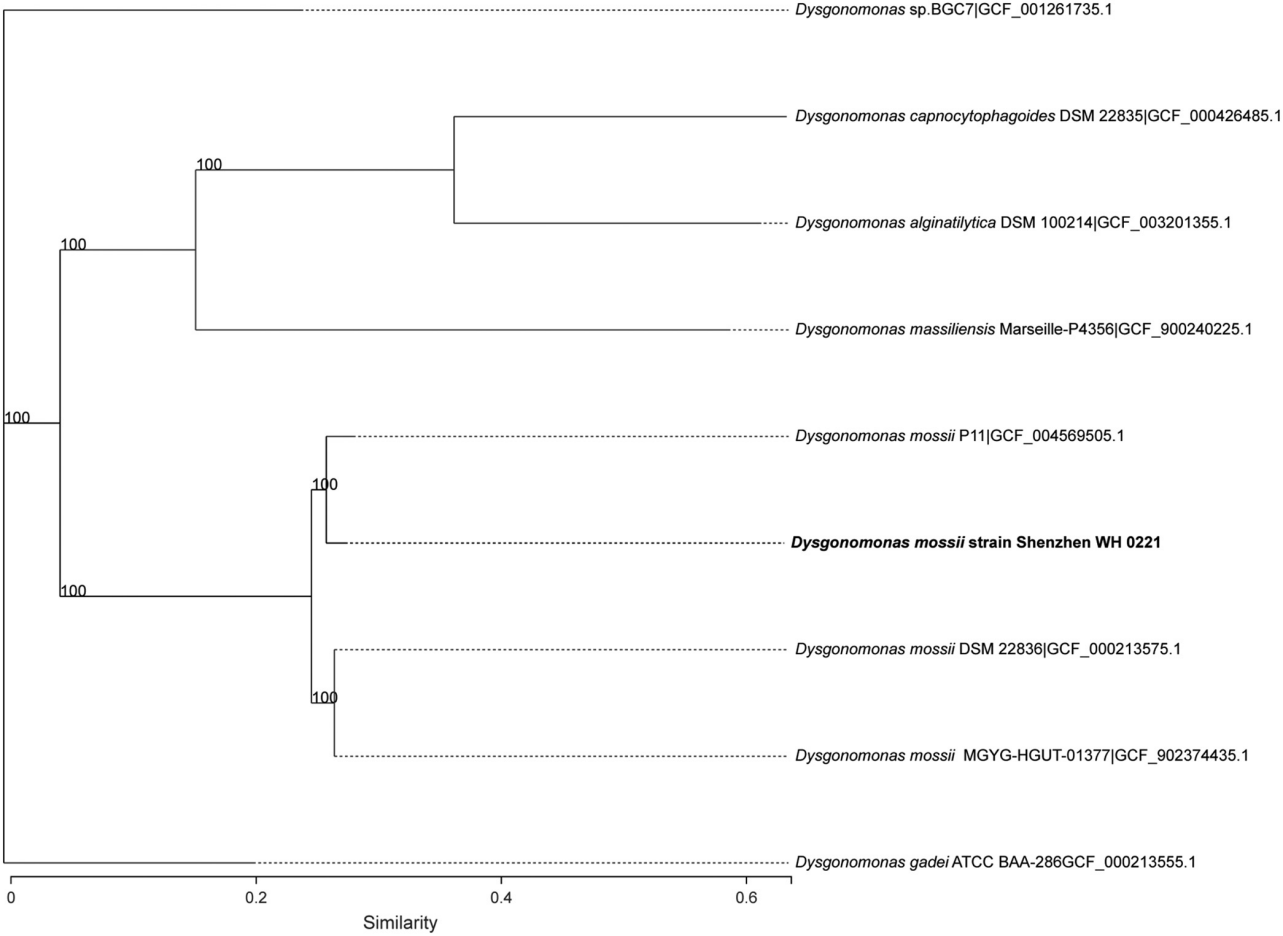
The phylogenetic tree based on single copies of homologous genes was constructed with model prediction software jModelTest and Bayesian Information Criterion with 1,000 bootstrap iterations. Each branch of the tree represents a species, and the length of the branch is the evolutionary distance between two species.

Eight genome sequences from the genus *Dysgonomonas* were downloaded from NCBI. General features of *Dysgonomonas* genome sequences are described in Table 1. Then, a phylogenetic tree based on single copies of homologous genes was constructed (Fig. 5). As shown in Figure 5, Shenzhen WH 0221 was most related to Dysgonomonas mossii. Additionally, average nucleotide identities (ANI) between the genome sequences of the eight strains and the sequence of strain Shenzhen WH 0221 ranged from 83.6 to 96.0% (Table 2). The ANI values between Shenzhen WH 0221 and Dysgonomonas mossii DSM P11 (from mouse oral tissue), Dysgonomonas mossii DSM 22836 (from a biological product), and Dysgonomonas mossii DSM MGYG-HGUT-01377 (from human gut) were 96.0%, 95.2%, and 95.2%, respectively. The accepted threshold for species definition is 95%. Therefore, the novel organism isolated from the blood of a patient with underlying diseases was identified as a member the species of Dysgonomonas mossii. We named it Dysgonomonas mossii strain Shenzhen WH 0221.

**TABLE 2:** Average nucleotide identity (ANI) between each two genomes

Species	GenBank no.	Shenzhen WH P0221 ANI (%)
Dysgonomonas mossii DSM P11	GCF_004569505.1	96.0
Dysgonomonas mossii DSM 22836	GCF_000213575.1	95.2
Dysgonomonas mossii DSM MGYG-HGUT-01377	GCF_902374435.1	95.2
Dysgonomonas capnocytophagoides DSM 22835	GCF_000426485.1	87.3
Dysgonomonas gadei ATCC BAA-286	GCF_000213555.1	86.5
*Dysgonomonas* sp. BGC7	GCF_001261735.1	85.0
*Dysgonomonas massiliensis* Marseille-P4356	GCF_900240225.1	84.3
Dysgonomonas alginatilytica DSM 100214	GCF_003201355.1	83.6

### Physiological and biochemical characterizations.

Based on the Vitek 2 ANC card, the strain Shenzhen WH 0221 can utilize d-galactose, d-biodisaccharide, d-glucose, d-mannose, d-maltose, sucrose, *N-acetyl-d*-glucosamine, 5-Bromo-4-chloro-3-indole-β-d-glucoside, β-galactose pyranosidase indoxyl, maltotriose, 5-Bromo-4-chloro-3-indole-α-d-mannose glycoside, and arbutin and gave positive results for alanine-phenylalanine-proline arylamine enzyme, β-mannosidase, and phosphatase. ANC card analysis also demonstrated that Shenzhen WH 0221 is Gram-negative, bacillary, and facultatively anaerobic. All other ANC tests were negative. Then, the Vitek 2 GN card revealed that Shenzhen WH 0221 can also degrade Palatinose, d-mycose, and secreted β-galactosidase, β-N-Acetylglucoside, β-glucosidase, lipoidase, and α-galactosidase. Characteristics differentiating the strain Shenzhen WH 0221 from other members of the genus *Dysgonomonas* are shown in Table 3. Members of the genus *Dysgonomonas* compared were most commonly isolated from human sources, such as abdominal wounds, the gallbladder, and abdominal drains. Based on these biochemical characterizations, Shenzhen WH 0221 can be distinguished from Dysgonomonas mossii CCUG 43457^T^ based on sugar fermentation types and major fatty acids. Shenzhen WH 0221 can utilize trehalose and sucrose, but not L-arabinose, while Dysgonomonas mossii CCUG 43457^T^ has the opposite capacity (Table 3).

**TABLE 3:** The different characteristics of strain Shenzhen WH 0221 and other members of the genus *Dysgonomonas*^*a*^

Characteristic	1	2	3	4	5	6	7
	Human clinical sample						
Isolation source	Blood	Abdominal wound	Stool samples	Gall bladder	Abdominal drain	Stool sample	Microbial fuel cell
Production of:							
β-Galactosidase	+	−	+	+	+	−	+
β-Glucuronidase	−	−	−	+	−	ND	+
N-acetyl-β- glucosaminidase	+	+	−	+	+	+	+
α-Fucosidase	−	+	−	+	−	ND	+/−
α-Arabinosidase	−	−	+	+	−	ND	+
Alkaline phosphatase	+	+	+	+	+	−	+
Fermentative growth on:							
L-Arabinose	−	−	+	+	+	+	+
Trehalose	+	+	−	+	−	ND	−
Sucrose	+	+	+	+	−	ND	+
Major fatty acid	C_14:0_ 3-OH, C_14:0_ 9-CH_3_, C_16:0_	anteiso-C_15:0_, iso- C_14:0_, iso C_16:0_ 3- OH	anteiso-C_15:0_, C_15:0_, anteiso- C_17:0_ 3-OH	anteiso-C_15:0_, C_16:0_, iso- C_14:0_	anteiso-C_15:0_, C_15:0_, iso- C_14:0_	antesio C_15:0_, C_15:0_, C_16:0_	anteiso C_15:0_, iso-C_17:0_ 3-OH, C_16:0_ 3-OH

a Strains: 1, Dysgonomonas mossii strain Shenzhen WH 0221(data from this study); 2, Dysgonomonas hofstadii CCUG 54731^T^ (1); 3, Dysgonomonas capnocytophagoides JCM 16697^T^ (2); 4, Dysgonomonas gadei CCUG 42882^T^ (1145589) (2); 5, Dysgonomonas mossii CCUG 43457^T^ (6); 6, *Dysgonomonas Marseille*-P4356^T^ (19); 7, Dysgonomonas oryzarvi Dy73^T^. ND, no data available; +, Positive; −, Negative.

### Composition of cellular fatty acids.

The overall fatty acids profile of Dysgonomonas mossii strain Shenzhen WH 0221 is shown in Table 4. The primary types include 3-hydroxy long-chain fatty acids (large amounts, about 44.3%), branched chain fatty acids and straight chain saturated (moderate amounts, about 16%), and unsaturated fatty acids and iso-methyl branched fatty acids (small amounts, about 10%). Compared to other species of genus *Dysgonomonas*, Shenzhen WH 0221 possesses a distinct fatty acid profile characterized by C_14:0_ 3-OH, C_14:0_ 9-CH3, and C_16:0_ (Table 3). The major types found in Dysgonomonas mossii CCUG 43457^T^ were anteiso-C_15:0_, C_15:0_, and iso- C_14:0_ (Table 3).

**TABLE 4:** Fatty acids profile of strain Dysgonomonas mossii strain Shenzhen WH 0221

Types of fatty acids	Fatty acids	Name	Mean relative (mean ± SD) %^*a*^	Total (Mean ± SD) %^*a*^
3-hydroxy long-chain fatty acids	C_14:0_ 3-OH	3-hydroxytetradecanoic acid	36.8 ± 1.8	44.3 ± 1.9
	C_16:0_ 3-OH	3-hydroxy-hexadecanoic acid	7.5 ± 0.5	
Branched chain fatty acids	C_14:0_ 9-CH_3_	9-methyltetradecanoic acid	16.8 ± 1.2	16.8 ± 1.2
Straight chain saturated	C_16:0_	Hexadecanoic acid	11.4 ± 0.5	16.5 ± 0.4
	C_18:0_	Octadecanoic acid	2.9 ± 0.2	
	C_15:0_	Pentadecanoic acid	2.2 ± 0.1	
Unsaturated fatty acids	△9,12-C_18:2_	9,12-Octadecadienoic acid	8.5 ± 0.6	10.8 ± 0.7
	△9-C_18:1_	9-Octadecenoic acid	2.4 ± 0.2	
Iso-methyl branched	iso-C_14:0_	13-methyltetradecanoic acid	5.1 ± 0.3	7.2 ± 0.5
	iso-C_13:0_	12-methyltridecanoic acid	2.1 ± 0.2	

a Mean peak area percentage; SD, standard deviation.

### Antibiotic resistance.

Next, we evaluated the characteristics of this microbe and its susceptibility to antimicrobial agents. In this study, Dysgonomonas mossii strain Shenzhen WH 0221 was isolated from the anaerobic blood culture bottles of a 78-year-old female with diabetes mellitus, hypertension, and pneumonia, as well as symptoms of hyperkalemia, serious osteoporosis, and nephrosis. The isolated strain demonstrated resistance to various cephalosporins (cefazolin sodium, ceftazidime, ceftriaxone), aminoglycosides (gentamicin, amikacin), β-lactams (penicillin), fluoroquinolones (norfloxacin), glycopeptides (vancomycin), and phenicols (chloramphenicol), but was susceptible to carbapenems (meropenem, imipenem), tetracycline, and clindamycin (Table 5). Compared with the other two reported strains of Dysgonomonas mossii (12, 19), MICs of Shenzhen WH 0221 differed for some antimicrobial agents, including higher MICs for penicillin and cefoperazone-sulbactam, median levels for chloramphenicol, and lower levels for ampicillin. The appropriate antibiotics for eradicating patient infection by Shenzhen WH 0221 were amoxicillin-clavulanic acid, ampicillin, meropenem, imipenem, and clindamycin. In fact, the female was infected with Enterobacter gergoviae, Klebsiella pneumoniae, and Stenotrophomonas maltophilia in the respiratory tract at the same time she developed sepsis. The patient was treated with intravenous injection of cefoperazone-sulbactam (a single dose of 3 g every 8 h) based antibiotic-susceptibility testing of K. pneumoniae. Inflammatory indexes (Fig. S1) decreased after 5–6 days of treatment (the day blood culture performed was defined as day 0), at which time blood culturing was negative. We hypothesize that Shenzhen WH 0221 was cleaned out from the blood by continuous use of cefoperazone-sulbactam (its MIC is 12 μg/mL) and improved immunity.

**TABLE 5:** Antimicrobial susceptibility test^*a*^

Antibiotic types	Antimicrobial agent	Shenzhen WH 0221		1 MIC (μg/mL)	2 MIC (μg/mL)
		Zone of inhibition (mm)	MIC (μg/mL)		
Aminoglycosides	Gentamicin	6.0	≥256	ND	ND
	Amikacin	6.0	≥256	ND	ND
β-lactams	Amoxicillin-clavulanic acid	ND	0.25	≤2	≤1
	Penicillin	ND	≥32	>1	ND
	Ampicillin	21.2 ± 1.9	1	>1	≥8
Carbapenems	Meropenem	21.6 ± 5.8	0.19	≤0.25	≤0.12
	Imipenem	31.7 ± 5.3	0.5	≤0.25	ND
Cephalosporins	Cefazolin Sodium	6.0	≥256	ND	ND
	Ceftriaxone	6.0	24	ND	≥4
	Ceftazidime	6.0	≥256	16	ND
	Cefoperazone-Sulbactam	21.4 ± 2.7	12	≤1	ND
Fluoroquinolones	Levofloxacin	10.4 ± 0.7	6	>2	≥4
	Norfloxacin	6.0	≥256	ND	ND
Tetracyclines	Tetracycline	ND	0.25	ND	≤0.5
Glycopeptides	Vancomycin	ND	≥32	ND	ND
Lincosamides	Clindamycin	ND	1	0.5	ND
Phenicols	Chloramphenicol	ND	6	16	2
Isolation Source		Blood of a patient with diabetic nephropathy		Blood of a patient with hepatocellular carcinoma	Intestinal juice of a patient with pancreatic cancer

a The experiment was repeated 3 times independently. ND, no data available. 1, Dysgonomonas mossii (19); 2, Dysgonomonas mossii (12).

To date, only three reports describe Dysgonomonas mossii infection in humans (6, 12, 19), of which only two investigated its drug-resistant phenotype. However, limited antibiotics were researched for the two previously reported strains. Seventeen antibiotics spanning at least 6 classes were studied, providing a valuable guide to the clinical usage of drugs to treat Dysgonomonas mossii infection.

### Analysis of drug resistance genes and virulence genes.

For the first time, we report genome-based functional predictions for Dysgonomonas mossii. Drug resistance genes were analyzed based on CARD. Nearly 100 resistance determinants are found in the isolate (data set in the supplemental material). Five antibiotic resistance ontologies (AROs) likely underlie the multidrug resistance of Shenzhen WH 0221 (Table 6). Efflux pump proteins and related genes are *kdpE* (20), *ykkD* (21), and *cmeB* (22), potentially explaining resistance to aminoglycoside, phenicol, cephalosporin, and fluoroquinolone antibiotics. TLA-3 is a β-lactamase (23). It confers resistance to ceftazidime, cefotaxime, and cefepime, but not to meropenem. *vanRM* is a vanR variant found in the vanM gene cluster and can confer resistance to vancomycin (24).

**TABLE 6:** Prediction of drug resistance genes based on CARD

Resistance mechanism	ARO^*a*^ name	Drug class
Antibiotic efflux	*kdpE*	Aminoglycoside
	*ykkD*	Aminoglycoside, Phenicol
	*cmeB*	Cephalosporin, Fluoroquinolone
Antibiotic inactivation	*TLA-3*	Cephalosporin, Fluoroquinolone, β-lactams
Antibiotic target alteration	*vanRM*	Glycopeptide

a ARO: Antibiotic Resistance Ontology.

The genome of strain Shenzhen WH 0221 carries more than 200 genes encoding putative virulence factors, including offensive virulence factors, defensive virulence factors, nonspecific virulence factors and regulators of virulence-associated genes (data set in the supplemental material). These genes probably play important roles in bacterial survival, persistence, and evasion of host immune response. Offensive virulence factor-like capsule (VF0323) increases bacterial adherence to host cells (25). Defensive virulence factors capsules (VF0144, VF0003) prevent activation of the alternative complement pathway and inhibit complement-mediated opsonophagocytosis (26). Lipopolysaccharides play a role in entry and early survival within macrophages, in addition to promoting resistance to innate-immunity antibacterial responses (27). Cytolysin is capable of lysing erythrocytes, polymorphonuclear leukocytes, and macrophages (28). The strain Shenzhen WH 0221 is predicted to be a human pathogen, especially in immunocompromised hosts, based on a combination of clinical inflammation symptoms and pathogenic systemic analysis of the genome.

## DISCUSSION

Generally, members of the genus *Dysgonomonas* have been found in environmental materials (7) and clinical samples (29). With the development of next-generation sequencing, more microorganisms from *Dysgonomonas* have been detected in complex microbiomes, such as periodontal microbiota (30), nasal surface samples (31), and the digestive tracts of several insects (10, 32) and *Eriocheir sinensis* (33). In the study, we identified a new strain of *Dysgonomonas* (named Dysgonomonas mossii strain Shenzhen WH 0221) based on phylogenetic analysis of 16S rDNA, housekeeping genes, and single-copy homologous genes from a blood sample of an immunocompromised patient who had been hospitalized for nearly 1 month. The strain Shenzhen WH 0221 was consistent with other species from the genus *Dysgonomonas* in morphology (coccobacillus-shaped organisms) and culture environment (facultative anaerobic). However, physiological and biochemical characterizations, as well as overall fatty acid profile, distinguish Shenzhen WH 0221 from other species of the same genus.

Three strains of Dysgonomonas mossii have been found from human sources so far. The first strain of Dysgonomonas mossii, CCUG 43457^T^, was isolated from the abdominal drainage fluid of a female patient with colon cancer in the United States in 2002 (6). The second was isolated from the intestinal juice of a patient with pancreatic cancer in Japan in 2006; however, its presence did not lead to significant clinical infection (12). The third, which caused bacteremia, was identified from a patient with cholangitis and hepatocellular carcinoma in Japan in 2018 (19). The strain Shenzhen WH 0221 represents the first Chinese case report of sepsis caused by Dysgonomonas mossii in a patient with diabetic nephropathy. These rare cases indicate that more attention should be paid to the isolation of this species and its possible roles in various infections. Due to limited research on its phenotypes and genomes, there is currently no standard treatment for Dysgonomonas mossii infection. Seventeen antibiotics spanning at least 6 classes were assessed, and genome analysis was performed in Dysgonomonas mossii strain Shenzhen WH 0221. We determined that it is resistant to most cephalosporins, gentamicin, amikacin, penicillin, norfloxacin, and vancomycin, but is susceptible to meropenem, imipenem, tetracycline clindamycin, and amoxicillin-clavulanic acid. Compared with the phenotypes of the other two Dysgonomonas mossii strains, all were sensitive to amoxicillin-clavulanic acid and meropenem. These findings support the use of amoxicillin-clavulanic acid and meropenem to eradicate Dysgonomonas mossii infection.

The pathogenic mechanism of Dysgonomonas mossii has not been reported. Strains of Dysgonomonas mossii isolated from clinical samples were all obtained from patients who were more than 60 years old and suffered severe underlying diseases or cancers, as described above. Some researchers have hypothesized that this species may be a normal component of intestinal flora that acts as an opportunistic pathogen (12, 19). We have analyzed the pathogenic system based on genome sequencing (first reported to-date for Dysgonomonas mossii). Among these predicted virulence factors and resistance determinants, lipopolysaccharides, capsules, and cytolysin were supposed vital to strain survival and evasion of host immunity systems; at least five genes (*kdpE*, *ykkD*, *cmeB*, *TLA-3* and *vanRM*) may be involved in its multidrug resistance. Many rare species cannot be identified in a timely manner in clinical practices, resulting in adverse outcomes. Therefore, more researchers should be encouraged to study these unknown potentially pathogenic bacteria, particularly with respect to their drug resistance and virulence factors.

## MATERIALS AND METHODS

### Strain isolation.

The new strain, Shenzhen WH 0221, was originally isolated from the blood of an immunocompromised patient. The blood was sampled and cultured in blood culture vials (Bactec 9120 SYSTEM, BD, USA) aerobically and anaerobically at 35°C for at least 5 days. Two sets (collected twice by venipuncture at different sites) of blood culture vials were cultured aerobically and anaerobically simultaneously. Two aerobic bottles were negative, while two anaerobic bottles flagged positive when cultured for 3 days. The isolate was obtained from anaerobic bottles after passage on Columbia Blood Agar (Berite Biotechnology, Zhenzhou, China) grown at 37°C for 1 day.

### MALDI-TOF MS.

Matrix-assisted laser desorption/ionization–time-of-flight mass spectrometry (MALDI-TOF MS) (Microflex LT/SH, Bruker, Germany) and IVD-Database Knowledge Version 8 (7854MSP, Bruker, Germany) were used to identify the bacterial strain. The strain Shenzhen WH 0221 was cultivated anaerobically on Columbia Blood Agar at 37°C for 1 day. Fresh bacterium was spotted on a target plate (96 polished ID101, Bruker, Germany) and covered first with 1 μL 70% formic acid, then with 1 μL IVD Matrix HCCA-portioned (Bruker, Germany). The sample was analyzed using the MALDI-TOF MS instrument following the manufacturer’s recommendations. Positive identification was defined as a confidence value ≥2.0.

### Phenotypic characterization.

Colony morphology was observed after anaerobic growth on Columbia Blood Agar at 37°C for 24 h using Anaeropack (Tokyo, Japan). Gram-staining of the harvest cells was carried out by using SMART Auto stainer (KS-S100 series, KOREA STANDARD, Korea). Then, an inverted microscope (Axio Observe 3, Carl Zeiss Miroscopy GmbH, Germany) was used to observed bacterial morphology under 1000× magnification. The size of cells and presence or absence of flagella were determined using transmission electron microscopy (TEM; Talos L120C, Thermo Fisher, USA). In brief, fresh bacterial cells were washed once with PBS, after which 10 μL bacterial solution was added to copper mesh and allowed to attach for about 10 min. After excess bacterial fluid was absorbed with filter paper, samples were stained for 1 min with 1% uranyl acetate dihydrate added to the copper mesh.

Biochemical characterization was performed using the Vitek 2 Anaerobic and Corynebacteria identification card (ANC) and GN card according to the manufacturer’s instructions (Vitek 2 COMPACT SYSTEM, Marcy l’Etoile, France).

### Cell fatty acid–fatty acid methyl ester analysis.

The strain Shenzhen WH 0221 was cultivated anaerobically on Columbia Blood Agar at 37°C for 1 day. Fresh cells were harvested and washed three times with water. Cellular fatty acids were methyl-esterized by the addition of sodium hydroxide-methanol solution, then allowed to react at 100°C for 1 h with shaking (800 rpm/minute). Samples were cooled at room temperature (25 ± 5°C), combined with hydrochloric acid-methanol solution, allowed to react at 80°C for 30 min, and immediately cooled on ice. Fatty acid methyl esters were extracted with petroleum ether three times for each sample. Supernatant solvent was evaporated to dryness under a stream of high-purity nitrogen. The residue was dissolved with normal hexane and analyzed by GC-MS (GC, Agilent 7890A, USA; MS, Agilent 5975C, USA).

### Antibiotic susceptibility testing.

Antimicrobial susceptibility tests were performed using both the Kirby-Bauer Disk Diffusion Method (K-B method) and the Etest method with Mueller–Hinton Blood Agar (Berite Biotechnology, Zhenzhou, China) following the manufacturer’s instructions. In brief, the strain was cultivated anaerobically on Columbia Blood Agar at 37°C for 1 day. The bacterial suspension was adjusted to 0.5 MCF (McFarland Standard; the McFarland suspension used was 0.45% NaCl), after which a sterile cotton-tipped swab was used to streak the entire agar surface three times, rotating the plate 60 degrees each time to evenly distribute the inoculum, and then disks of different antibiotics (K-B method) or Etest strips were attached to the surface of Mueller–Hinton Blood Agar as stated above. Inhibition zones were measured using a vernier caliper, and MICs were determined based on inhibition zones after 1 day of anaerobic culture at 37°C for 1 day. The antimicrobials used for susceptibility testing included 17 antimicrobial agents belonging to the following classes: aminoglycosides (gentamicin, amikacin), β-lactams (amoxicillin-clavulanic acid, penicillin, ampicillin), carbapenems (meropenem, imipenem), cephalosporins (cefazolin sodium, ceftriaxone, ceftazidime, cefoperazone-sulbactam), fluoroquinolones (levofloxacin, norfloxacin), tetracyclines (tetracycline), glycopeptides (vancomycin), lincosamides (clindamycin), and phenicols (chloramphenicol).

### Amplification of 16S r DNA and Sanger sequencing.

Total genomic DNA was extracted using the TIANamp Bacteria DNA Kit (DP302, TIANGEN, Beijing, China). Then, 16S rRNA gene regions were amplified and sequenced with universal primers (27F, 5′-AGAGTTTGATCMTGGCTCAG-3′and 1492R, 5′-GGTTACCTTGTTACGACTT-3′) according to previously published literature (34). PCR products were purified using an Agarose Gel DNA Clean-Up purification kit (DP208/209, TIANGEN, Beijing, China) and sequenced using the primer 27F and an ABI Prism 3730xl DNA analyzer system (USA). Homology comparison was performed through the National Center for Biotechnology Information (NCBI) website (https://blast.ncbi.nlm.nih.gov/Blast.cgi?PROGRAM=blastn&PAGE_TYPE=BlastSearch&LINK_LOC=blasthome).

### Phylogenetic analysis of 16S r DNA.

The closest known relatives of the isolates were screened using database searches on the NCBI website. Then, the sequences of related strains were retrieved from the NCBI website and aligned with the newly determined sequences using the program MEGA 6 (35). Multiple sequence alignment was corrected manually based on the regular algorithm, and a distance matrix was calculated by the program. According to the neighbor-joining method, a phylogenetic tree was constructed via bootstrap analysis (number of bootstrap replications: 1,000).

### Phylogenetic analysis of housekeeping genes.

Phylogenetic analysis of 31 housekeeping genes (*dnaG*, *frr*, *infC*, *nusA*, *pgk*, *pyrG*, *rplA*, *rplB*, *rplC*, *rplD*, *rplE*, *rplF*, *rplK*, *rplL*, *rplM*, *rplN*, *rplP*, *rplS*, *rplT*, *rpmA*, *rpoB*, *rpsB*, *rpsC*, *rpsE*, *rpsI*, *rpsJ*, *rpsK*, *rpsM*, *rpsS*, *smpB*, *tsf*) from 19 closely related species was performed to construct a phylogenetic tree using MEGA 6.0 software (35) based on the protocol described above.

### Whole genome sequencing and genome assembly.

Genomic DNA was extracted from the new strain using the Wizard Genomic DNA purification kit (Promega, Shanghai, China) according to the manufacturer’s protocol. Fragments of 150–500 bp were enriched by shearing DNA samples using a Covaris M220 Focused Acoustic Shearer (ThermoFisher Scientific, Waltham, MA, United States). Sequencing libraries were prepared from these sheared fragments with a NEXTflex Rapid DNA-Seq Kit (Bio Scientific, Austin, TX, USA). Paired-end sequencing of libraries with variant insert sizes (2 × 150 bp) was performed on the Illumina HiSeq X 10 platform (Majorbio Bio-pharm Technology, Shanghai, China).

Low-quality data were removed by quality trimming based on statistical analysis of quality information, generating clean data. Scaffolds were assembled from clean reads using the short sequence assembly software SOAPdenovo2 (36). Glimmer (http://ccb.jhu.edu/software/glimmer/index.shtml), tRNAscan-SE v2.0 (http://trna.ucsc.edu/software/), and Barrnap (https://github.com/tseemann/barrnap) were used to predict coding sequences (CDSs), tRNAs, and rRNA, respectively, from the genome. Functional annotations were generated by comparison with NR, Swiss-Prot, Pfam, EggNOG, GO, and KEGG databases. Gene annotations were primarily based on protein sequence comparison. Gene sequences were compared to each database to obtain the best-matched subject (E value ≤1e-5). A circular genomic plot of the strain was generated by Circos (37).

### Analysis of drug resistance genes and virulence genes.

The genomic sequence of Dysgonomonas mossii strain Shenzhen WH 0221 was assessed for resistance genes and virulence genes by DIAMOND (38) based on the Comprehensive Antibiotic Resistance Database (CARD) (http://arpcard.Mcmaster.ca, Version 1.1.3) and Virulence Factors of Bacterial Pathogens (VFDB) database (39), respectively. Query sequences were compared to the CARD or VFDB database, and the default threshold was set as E value ≤1e-5.

### Comparative genomic analysis.

Genomic data from several species of genus *Dysgonomonas* were obtained from the NCBI. Single copies of homologous genes were multiply aligned using IQ-TREE (ML) software. Phylogenetic trees were constructed using model prediction software jModelTest and Bayesian information criterion (BIC) with 1,000 bootstrap iterations (40). To compare the genetic relationships among members of the genus *Dysgonomonas*, average nucleotide identity (ANI) values were calculated using pyani (41).

### Data availability.

The new strain, Dysgonomonas mossii strain Shenzhen WH 0221, was named after the city (Shenzhen, China), the family names of the finders (Jiehong Wei and Tongyu Hao), and the date of discovery (February 21). The genome sequence has been deposited at GenBank and Sequence Read Archive (SRA) under the accession numbers JAMWSN000000000 and SRR19882120, respectively. Complete sequence of the 16S rRNA gene has been deposited in GenBank under the accession number OK254132.
