# Supplementary material for: Dysgonomonas mossii Strain Shenzhen WH 0221, a New Member of the Genus Dysgonomonas Isolated from the Blood of a Patient with Diabetic Nephropathy, Exhibits Multiple Antibiotic Resistance
Source: Microbiol Spectr. 2022 Aug 1;10(4):e02381-21. doi: 10.1128/spectrum.02381-21 (PMC9431661; doi:10.1128/spectrum.02381-21)
Supplement: Supplemental file 1 — Supplemental material. Download spectrum.02381-21-s0001.pdf, PDF file, 0.2 MB [file spectrum.02381-21-s0001.pdf]

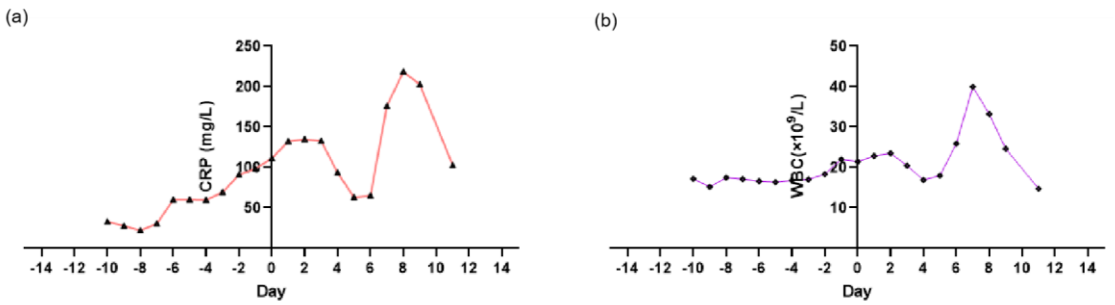

Fig. S1. Daily changes in CRP and WBC count. a, CRP variation before and after 10 days of positive blood cultures. b, Changes in WBC detected before and after 10 days of positive blood culture.

Table S1. Sequence similarity values > 99.5% of the 16S rRNA gene sequence (1,363bp) of strain Shenzhen WH 0221 with type strains using Blast engine

Number	Species	GenBank Number	Identity (%)	Source
1	*Dysgonomonas oryzarvi* strain CBA7536	MN646999	99.71	Geum River
2	*Dysgonomonas* sp. AM15	EU252503	99.71	East China Sea
3	*Dysgonomonas* sp. WJDL-Y1	KF176996	99.63	ND
4	*Dysgonomonas mossii* strain P11-biofilm	MW175551	99.63	ND
5	*Dysgonomonas* sp. A1	HQ659694	99.63	Sludge

ND, no data available

Table S2. Summary of sequenced libraries for Shenzhen WH 0221 after filtering and genome mapping

Summary	*Dysgonomonas mossii* Shenzhen Wh0221
Raw Pair Reads	5573238×2
Clean Pair Reads	5573238×2
Clean bases (bp)	1.7E+09
Raw Q20 (%)	97.27
Raw Q30 (%)	93.21
Clean Q20 (%)	97.41
Clean Q30 (%)	92.42

Table S3. Summary details based on housekeeping genes when compared with Shenzhen WH 0221

GenBank Number	Species	Identity (%)	Coverage(%)
GCF_004569505.1	*Dysgonomonas mossii* P11	99.8	100
GCF_000213575.1	*Dysgonomonas mossii* DSM 22836	99.4	100
GCF_001261735.1	*Dysgonomonas sp. BGC7*	92.6	100
GCF_000213555.1	*Dysgonomonas gadei* ATCC BAA-286	92.4	100
GCF_000711235.1	*Prevotella* sp. 10(H)	92	100
GCF_900343125.1	*Dysgonomonas sp. Marseille*-P4361	90.9	100
GCF_000426485.1	*Dysgonomonas capnocytophagoides*	86.2	100
GCF_900128985.1	*Dysgonomonas macrotermitis*	86	100
GCF_001261715.1	*Dysgonomonas* sp. HGC4	85.5	100
GCF_003201355.1	*Dysgonomonas alginatilytica*	85.4	100
GCF_900240225.1	*Dysgonomonas massiliensis*	85.5	100
GCF_002849245.1	Dysgonamonadaceae bacterium	78.7	99.97
GCF_900095795.1	*Petrimonas mucosa*	78.6	99.97
GCF_900178525.1	*Massilibacteroides vaginae*	78.7	100
GCF_900162725.1	*Parabacteroides sp.* Marseille P3160	77.6	100
GCF_000803105.1	*Coprobacter secundus*	78.2	100
GCF_000473955.1	*Coprobacter fastidiosus*	78.2	100
GCF_001282625.1	*Lascolabacillus massiliensis*	77.6	99.97
GCF_001657575.1	*Bacteroidales* bacterium Barb4	77.1	100
